# Career coaching to support medical student career decision-making: a randomized controlled trial

**DOI:** 10.1007/s10459-025-10409-8

**Published:** 2025-03-03

**Authors:** Daan A. H. Fris, Annelies E. M. van Vianen, Edwin A. J. van Hooft, Matthijs de Hoog, Anne P. J. de Pagter

**Affiliations:** 1 Department of Pediatrics, Erasmus Medical Center - Sophia Children’s Hospital, Rotterdam, The Netherlands; 2 Work and Organizational Psychology, University of Amsterdam, Amsterdam, The Netherlands; 3 Work and Organisational Psychology, Open University, Heerlen, The Netherlands; 4 Department of Neonatal & Pediatric Intensive Care, Division of Pediatric Intensive Care, Erasmus Medical Center – Sophia Children’s Hospital, Rotterdam, The Netherlands; 5 Department of Pediatrics, Division of Stem Cell Transplantation, Willem-Alexander Children’s Hospital, Leiden University Medical Center, Leiden, The Netherlands; 6 Department of Quality and Patient Safety, Leiden University Medical Center, Leiden, The Netherlands; 7 Department of Quality and Patient Safety, Erasmus Medical Center Rotterdam, Rotterdam, The Netherlands

**Keywords:** Career coaching, Career choice, Specialty choice, Medical students, Career decision-making stress

## Abstract

This study investigated whether career coaching can reduce medical students’ career decision-making stress through clarifying students’ self-concept, increasing career decision self-efficacy, and lowering perceived time pressure to make a career decision. We evaluated the effectiveness of a coaching intervention (including five individual coaching sessions over eight months) using a randomized-waitlist controlled trial design. Participating medical master’s students completed a first survey (T1). The intervention condition (*n* = 94) started the coaching program right away. The waitlist-control condition (*n* = 130) started after 8.5 months. Participants in the intervention condition completed a second (T2) survey three weeks after their last coaching session. Participants in the waitlist-control condition received the second survey 8.5 months after registration. Data were analyzed using multilevel path modelling. The total effect of coaching on career decision-making stress was significantly negative (-0.17; 95% CI [-0.31, -0.06]), reflecting a small to moderate effect size. This total effect was partially mediated (i.e., explained) by career decision self-efficacy (-0.02; 95% CI [-0.06, -0.00]). Exploratory analyses suggested that coaching only clarified the self-concept of students with a relatively negative coaching attitude at T1. Moreover, coaching resulted in more changes in career choices and an increase in students’ career choice certainty. This study demonstrates that coaching is effective in reducing medical students’ career decision-making stress and increasing their career decision self-efficacy and career choice certainty. High-quality career decision-making is important because it is associated with higher job satisfaction, well-being, and performance. Medical schools may consider providing coaching to students to support their career decision-making.

**Supplementary Information:**

The online version contains supplementary material available at 10.1007/s10459-025-10409-8.

Medical students in the master’s phase experience stress in choosing a future career direction (De Geneeskundestudent, 2019; Fris et al., 2022), outside or within medical care (Prismant, 2019). Career decision-making stress refers to “undesirable stress experienced during the career choice process” (Kleine et al., 2021; Stumpf et al., 1983) and is associated with a multitude of negative outcomes such as career indecision, negative affect, and lower psychological well-being (Arslan & Bayraktar-Uyar, 2020; Creed et al., 2016; Germeijs et al., 2006). The prevalence of career decision-making stress and the scarce research on career support for medical students highlight the need to evaluate the effectiveness of interventions aimed at reducing students’ career decision-making stress.

Prior studies on career interventions for medical students have primarily focused on influencing the direction of students’ career choices (e.g., Kozar et al., 2003; Pfarrwaller et al., 2015), while career support interventions often take a guiding approach or consist of multiple components (e.g., seminars and mentoring; Macaulay et al., 2007; Yoo et al., 2015; Zink et al., 2007). More recently, individual coaching has gained popularity as an intervention tool to facilitate the undergraduate to graduate medical education transition (Park et al., 2024; Winkel et al., 2023; Wolff et al., 2019). Coaching can be defined as a result-oriented, systematic process in which the coach facilitates the enhancement of life experience and goal attainment in the personal and/or professional life of non-clinical clients (Grant, 2003). Coaching focuses on self-directed change (Theeboom et al., 2017), where the client determines the goals to be attained. The coach guides the process rather than providing advice or information. This tailored individual approach differs from career workshops which are typically developed for a specific purpose and provided in a standardized manner to a larger group of students (Dik & Steger, 2008; Van der Horst et al., 2021). Unlike mentoring, coaching is not restricted to the client’s professional domain and coaches are trained professionals rather than senior colleagues (Fowler & O’Gorman, 2005). Also, coaching differs from therapy in that coaching focuses on non-clinical clients (Grant, 2003). Generally, coaching has been demonstrated to have favorable effects such as improvement in coping and well-being (Jones et al., 2015; Solms et al., 2021; Theeboom et al., 2013). To date, however, we do not know whether coaching is also beneficial to support medical students in career decision-making and reduce career decision-making stress.

This study investigates whether coaching is effective in reducing career decision-making stress among medical students during their clinical clerkships. Moreover, this study contributes to our theoretical understanding of the mechanisms involved in reducing career decision-making stress through coaching. This study provides important insights for practice on how to support medical students’ career decision-making.

## Theoretical framework

Medical education programs typically consist of several years of pre-clinical education and a period of clinical training, in which medical students gain practical work experience in various specializations (clinical rotations). These experiences allow them to further explore career directions and personal career preferences. Yet, students’ transition from pre-clinical education to the clinical setting often comes with undesirable stress about their future careers choices (De Geneeskundestudent, 2019). During the clerkships, students may for example discover that they dislike working as a clinician or that their initial specialty choice does not match their expectations, which may cause career decision-making stress.

Based on the beneficial outcomes of professional coaching in work contexts (Solms et al., 2021; Theeboom et al., 2013), we expect that coaching could support students in the career decision-making process. Specifically, we expect coaching to reduce career decision-making stress in three ways: by clarifying students’ self-concept, by increasing students’ career decision self-efficacy, and by reducing students’ perceived time pressure to make career decisions. Developing self-concept clarity is fundamental for making career choices and career coaching may assist in developing this clarity (Adam et al., 2018; Ebner, 2021; Hur et al., 2018). Likewise, career decision self-efficacy is a key factor in career decision-making (Choi et al., 2012) and raising clients’ self-efficacy is a central aim of (career) coaching (Theeboom et al., 2017). Finally, as students advance through their studies, students are aware that they will approach a point where they must make concrete decisions about their career path. This can result in perceptions of time pressure which is associated with higher stress (Fris et al., 2022; Wanberg et al., 2020). Coaching may help students to navigate and manage these perceptions effectively.

### Self-concept clarity

Self-concept clarity refers to ‘clearly and confidently defined, internally consistent, and temporally stable’ self-beliefs (Campbell et al., 1996, p. 141). A clear self-concept serves as a compass in career decision-making because potential career directions can be compared and contrasted with elements of this self-concept. Indeed, the clarity of these (vocational) self-beliefs is associated with greater career decidedness (Adam et al., 2018) and less career decision-making stress (Praskova et al., 2015).

Coaching can be helpful to stimulate reflection on one’s self concept (Rank & Gray, 2017; Theeboom et al., 2017). For example, coaches can stimulate clients’ self-reflection by asking thought-provoking questions (Terblanche, 2020). In addition, coaching offers an opportunity to look at one’s life from a broader perspective such as one’s (preferred) work-life balance. This will help to see how activities in life and work are related, fostering self-learning (Du Toit, 2007). Coaching allows medical students to reflect on their drives, values, and motivation (Kiuru et al., 2021; Whiston et al., 2017), personal life, clerkship experiences, and the interrelation between them. The structured process of self-reflection likely results in a more clearly defined self-concept, which in turn reduces career decision-making stress.

Prior coaching studies among a heterogeneous group of (postgraduate) students and medical students suggest that coaching may foster self-learning and understanding (Grant, 2014; Hur et al., 2018; Zhang et al., 2023). However, because these studies did not have a randomized control group, we do not know whether coaching caused these effects (Grant, 2014; Hur et al., 2018). As self-concept clarity tends to increase during early adulthood (Lodi-Smith & Roberts, 2010), it is possible that study participants naturally learned more about themselves over time, regardless of whether they were coached or not. Randomized controlled trials (RCT) aim to rule out alternative explanations such as maturation and selection. As a RCT design can show the value of coaching for developing self-concept clarity and reducing career decision-making stress, the present study used this more robust methodology.

### Career decision self-efficacy

Coaching could also reduce career decision-making stress through enhancing career decision self-efficacy. Career decision self-efficacy refers to the degree to which individuals feel confident at fulfilling tasks associated with career exploration and selection (Creed et al., 2006). More self-efficacious individuals appear to suffer less career indecision and career decision-making stress than less self-efficacious individuals (Choi et al., 2012; Fris et al., 2022).

Coaching may enhance self-efficacy through encouragement (verbal persuasion; Bandura, 1977) and reflection on successful experiences in the past (mastery experiences, Bandura, 1977; Betz et al., 1996; Van Zyl et al., 2016). Applying Bandura’s theorizing on self-efficacy (2021), coaches can compliment students on aspects of the career decision-making process that are going well, thereby increasing students’ confidence in their ability to successfully fulfill the tasks needed for making a career decision. Also, coaches can stimulate reflection on successful experiences during career decision-making processes in the past, such as students’ choice to study medicine. Coaches’ encouraging behaviors likely increase students’ confidence in career decision-making and therefore reduce career decision-making stress.

### Time pressure

Finally, we expect that coaching will reduce career decision-making stress through decreasing students’ perception of time pressure. Experiencing time pressure in making career decisions is strongly related to career decision-making stress (Fris et al., 2022). Challenging and reframing clients’ views, perspectives, and assumptions is one of the techniques coaches use to promote change in clients (Du Toit, 2007; Terblanche, 2020; Van Zyl et al., 2016). Coaches can challenge students’ beliefs about having to make a career decision on a short notice. For example, they could stimulate students to explore why they experience time pressure and whether their time perception is realistic or helpful. Coaches and students can also explore useful steps in the career decision-making process and make a realistic schedule for completing these steps. These activities may help improve time control and reduce perceived time pressure, which in turn reduce students’ career decision-making stress.

### Exploratory questions

In addition to testing the proposed research model, we explored whether the effects of coaching depend on students’ initial expectations of coaching. Furthermore, we explored if and how coaching affects students’ actual career choices (i.e., whether they change their initial choice options) and career choice certainty. These analyses increase our understanding of how coaching affects students’ career development in the transition to work. Specifically, it is a first step towards understanding how coaching may affect the quality of students’ transition to work. Practically, in the light of interventions attempting to steer medical students’ career choice (Kozar et al., 2003; Pfarrwaller et al., 2015), it is interesting to explore whether coaching affects their choices in a more self-directed manner.

### Study context

In 2021, a large Dutch university hospital initiated an individual coaching program to support medical students in career decision-making. The Dutch medical curriculum generally consists of a three-year bachelor’s and a three-year master’s phase. In the bachelor’s phase students attend classroom teaching and in the master’s phase students do clinical clerkships (i.e., in various medical specialties) in which they gain practical experience. After obtaining their master’s degree, students can work as licensed doctors and/or apply for a residency position.

## Method

### Coaching program and study population

The coaching program offered first-year medical master’s students a free coaching program. In the participating medical center, students start the master’s phase in cohorts of around 80 students. We conducted an a priori power analysis in G*Power. The results indicated that a minimum sample size of 159 participants was necessary to detect a small to medium effect size (f² = 0.1) with a significance level of α = 0.05. Taking into account potential non-response and dropout, we invited all students from six cohorts between February 2021 and February 2022 (*N* = 497) to participate in the study.

The coaching program included five individual coaching sessions of 1 to 1.5 h. Coaches and students were instructed to complete the coaching in approximately 8 months. The coaching sessions did not follow a specific protocol but were tailored to students’ needs and goals, in accordance with the definition of coaching (Grant, 2003; Theeboom et al., 2017). The program was not aimed at changing students’ career choices, nor were coaches aware of the specific focus of our study. Participating coaches were physicians who had received coach-training and were registered at the Dutch association of professional coaches (NOBCO). These coaches are trained to follow a solution-focused goal-oriented structure (the GROW method; Whitmore, 2010) throughout their coaching in which they focus on the client’s goal (What does the client want to achieve?), reality (Where is the client now?), obstacles and options (Which obstacles and options are there?), and way forward (What choices need to be made?).

Students were informed about the coaching program through a presentation before the start of their first clinical clerkship. During this presentation, students received information on what coaching is, the research they could participate in, and how they could sign up. Students registered voluntarily for a coaching program via a website. During registration, they could indicate whether they were willing to participate in the current study. Their participation was voluntary, i.e., students who declined participation could still sign up for coaching. Regardless of whether students participated in the study or not, the website randomly assigned students to the intervention condition or the waitlist control condition (see *design and procedure*) using a random number generator. The outcome of the randomization process was accessible only to the program coordinator, who informed students when they could start their program. Then, students could choose a coach based on introductory videos that the coaches provided and schedule a first appointment with their chosen coach. After the first session, participants could decide to switch coaches if they wished. Students were free to plan the sessions whenever they wanted, in consultation with their coach.

### Design and procedure

We used a randomized waitlist-controlled trial design: participants were randomly assigned to the intervention condition or the waitlist-control condition. We did not select nor stratify participants based on individual characteristics. The intervention condition started with the coaching program right away. The waitlist-control condition started after 8.5 months.

Upon approval from the ethical review board (IRB no. 2021-WOP-13117), data collection started. The study coordinator sent students a link to the Time 1 (baseline-measurement) survey by e-mail. Onto opening the link, students were informed about the purpose of the study, their right to withdraw at any given moment, and the confidentiality of their data. Then, participants gave their informed consent and started the survey. After having finished the first survey, the program coordinator informed students about the condition to which they were randomly assigned.

To assess the effectiveness of the coaching intervention, we administered a Time 2 measurement. Participants in the intervention condition received the Time 2 survey three weeks after their last coaching session (aimed to be 8.5 months after Time 1). The three-week period was chosen to ensure that students’ affective state after their last coaching session did not influence the measurement (Grant & O’Connor, 2018). To match the Time 2 measurement with the intervention group, participants in the waitlist-control condition received the Time 2 survey 8.5 months after registration. After completing the Time 2 survey, they could start with their coaching program. Completion of each questionnaire was rewarded with a €5,- gift card.

### Measures

All variables were measured at both Time 1 and 2. Self-concept clarity was measured with four items by Campbell et al. (1996). This scale has been demonstrated to be reliable and valid (Campbell et al., 1996; Lodi-Smith & Roberts, 2010). Career decision self-efficacy was assessed with three items from the reliable goal selection subscale of the Career Decision Self-Efficacy Scale (Betz et al., 1996). Time pressure was measured with four items from Fris et al. (2022) adapted from the Job Search Time Pressure scale (Wanberg et al., 2020). Career decision-making stress was measured with the four-item scale of Fris et al. (2022). Fris and colleagues (2022, 2024) found the time pressure and career decision-making stress scales to be reliable. The complete list of items is available as Online Resource 1. Cronbach’s alphas are presented in Table 1.

**Table 1:** Means, standard deviations, cronbach’s alphas, and correlations among study variables (N varies between 207 and 224)

	Total sample		Intervention condition		Waitlist-control condition																
Variable	M	SD	M	SD	M	SD	1	2	3	4	5	6	7	8	9	10	11	12	13	14	15
*Time 1*																					
1. Age	22.85	1.82	22.68	1.57	22.98	1.98	-														
2. Gender^a^	0.83	0.38	0.89	0.31	0.78	0.42	− 0.25**	-													
3. Coaching attitude	4.12	0.51	4.03	0.53	4.19	0.49	− 0.04	0.19**	(0.63)												
4. Condition^b^	0.42	0.49	-	-	-	-	− 0.08	0.15*	− 0.15*	-											
5. Self-concept clarity	3.32	0.77	3.24	0.80	3.38	0.75	− 0.06	− 0.03	0.08	− 0.09	(0.81)										
6. Career decision self-efficacy	3.51	0.67	3.50	0.69	3.51	0.67	− 0.01	0.03	0.16*	− 0.01	0.22**	(0.75)									
7. Time pressure	2.81	0.84	2.82	0.86	2.81	0.82	0.17*	0.02	− 0.02	0.01	− 0.11	− 0.14*	(0.87)								
8. Career decision-making stress	2.92	0.86	3.00	0.96	2.85	0.77	0.07	0.05	− 0.04	0.09	− 0.20**	− 0.21**	0.46**	(0.88)							
9. Career choice certainty	2.77	1.02	2.66	1.10	2.86	0.94	0.10	− 0.08	− 0.03	− 0.10	0.06	0.30**	− 0.05	− 0.14*	-						
*Time 2*																					
10. Self-concept clarity	3.48	0.70	3.51	0.73	3.46	0.69	− 0.07	0.04	− 0.00	0.04	0.66**	0.18**	− 0.11	− 0.18**	0.08	(0.76)					
11. Career decision self-efficacy	3.63	0.66	3.79	0.56	3.51	0.71	− 0.04	− 0.03	0.07	0.21**	0.31**	0.50**	− 0.09	− 0.15*	0.13	0.36**	(0.78)				
12. Time pressure	3.19	0.78	3.19	0.88	3.19	0.70	0.03	0.02	− 0.00	0.00	− 0.11	− 0.16*	0.29**	0.24**	− 0.07	− 0.18**	− 0.21**	(0.82)			
13. Career decision-making stress	3.04	0.91	2.91	0.91	3.13	0.84	0.05	0.11	0.03	− 0.12	− 0.09	− 0.17*	0.23**	0.40**	− 0.06	− 0.26**	− 0.29**	0.53**	(0.90)		
14. Career choice change^c^	0.56	0.50	0.67	0.47	0.48	0.50	0.09	− 0.06	0.02	0.19**	− 0.11	− 0.06	0.03	0.01	− 0.24**	− 0.17*	− 0.01	0.02	0.05	-	
15. Career choice certainty	3.12	0.98	3.27	0.95	3.02	0.99	0.01	− 0.10	0.00	0.13	0.03	0.33**	0.02	0.00	0.44**	0.06	0.45**	0.01	− 0.09	− 0.19**	-

Note *p <.05, **p <.01. Cronbach’s alphas are presented between brackets on the diagonal. Abbreviations: ^a^0 = male, 1 = female. ^b^0 = waitlist-control group, 1 = intervention group. ^c^0 = no, 1 = yes

### Exploratory measures

Coaching attitude was measured at Time 1 with three items from Solms et al. (2021). Current career choice and choice certainty were measured at both Time 1 and 2. Items are available in Online Resource 1. Changes in career choice were content analyzed and coded.

### Control variables

Age (T1), gender (T1), number of face-to-face and online coaching sessions (T2), and additional individual guidance (T2) were measured as potential control variables. Age was included as it positively relates to career decidedness (Neice & Bradley, 1979; Toyokawa & DeWald, 2020). Gender was included as women tend to worry more than men (Robichaud et al., 2003) and experience more career decision-making stress (Fris et al., 2022). As data were partially gathered during the COVID-19 pandemic, part of the coaching conversations took place in an online setting. We measured coaching setting as it might influence the effectiveness of coaching. Of the five coaching sessions, students had 1.32 conversations in person (*SD* = 1.93). Finally, we included additional (career) guidance as potential control variable because support is an important resource in career decision-making (Kleine et al., 2021; Whiston et al., 2017). We asked whether participants in the waitlist-control and intervention group had experience with individual guidance and/or coaching (next to the coaching offered in the current study) in the past 8 months (answer options included: no, a study advisor, a mentor, a coach or student psychologist, career guidance, and/or other). We created a binary variable distinguishing students who had received extra guidance (1) from those who had not (0).

We included or excluded control variables based on their correlations with condition and T2 career decision-making stress.

### Statistical analyses

We analyzed our data in Mplus 8.8 (https://www.statmodel.com/), as this allowed for testing the full path model while controlling for the nesting of participants within coaches. First, we examined the measurement invariance between the intervention and waitlist-control group at T2, which provided support for (at least) the metric invariance of our scales (see Table 2). Then, we tested our research model using multilevel path modelling to address the hierarchical structure of our data. Specifically, participants (defining Level 1) in the intervention group were grouped (defining Level 2) according to the coaches they had received coaching from (ranging from 1 to 7). The participants in the control group were each assigned their own individual cluster (ranging from 8 to 137). This was done because observations from the waitlist-control group were independent from each other (i.e., they were not affected by a common factor such as a coach). Controlling for clustering in this manner ensures that differences between the intervention group and the waitlist-control group can be more confidently attributed to the coaching intervention itself, rather than to variance between coaches.

**Table 2:** Measurement invariance between the intervention and waitlist-control condition at time 2 (N varies between 223 and 224)

Variable (T2)	Model	χ2 (df)	CFI	RMSEA	SRMR	Model comparison	Δχ2 (Δdf)	Decision
Self-concept clarity	Configural invariance	7.65 (4)	0.98	0.09	0.03			
	Metric invariance	11.31 (7)	0.98	0.07	0.06	Metric vs. configural	3.66 (3)	Accept
	Scalar invariance	17.29 (10)	0.97	0.08	0.09	Scalar vs. Metric	5.98 (3)	Accept
Career decision self-efficacy	Configural invariance	0 (0)	1	0	0			
	Metric invariance	2.61 (2)	1.00	0.05	0.08	Metric vs. configural	2.61 (2)	Accept
	Scalar invariance	17.35** (4)	0.93	0.17	0.13	Scalar vs. Metric	14.74** (2)	Reject
Time pressure	Configural invariance	14.93**(4)	0.97	0.16	0.04			
	Metric invariance	19.39** (7)	1.0	0.13	0.08	Metric vs. configural	4.46 (3)	Accept
	Scalar invariance	25.03** (10)	0.96	0.12	0.09	Scalar vs. Metric	5.64 (3)	Accept
Career decision-making stress	Configural invariance	12.52* (4)	0.99	0.14	0.02			
	Metric invariance	13.18 (7)	0.99	0.09	0.03	Metric vs. configural	0.67 (3)	Accept
	Scalar invariance	14.48 (10)	0.99	0.06	0.03	Scalar vs. Metric	1.29 (3)	Accept

Note *p <.05, **p <.01. The table presents our evaluation of configural, metric, and scalar measurement invariance of our variables of interest between the intervention and waitlist-control group at the T2 measurement. Configural invariance is the least stringent and tests whether the patterns of factor loadings are similar across groups. If support for configural invariance is found, metric invariance is tested. Metric invariance tests whether the factor loadings of each item on a factor are similar across groups. It is tested by constraining factor loadings to be equal across groups. Finally, when support for metric invariance is found, scalar invariance is tested, which is the most stringent. Scalar invariance test whether item intercepts are equivalent by constraining them to be equal across groups (Putnick & Bornstein, 2016). Overall, the results support at least metric invariance between the intervention and waitlist-control condition

Using path analysis we estimated relations between condition (0 = waitlist-control group, 1 = intervention group) and the mediators at T2 (self-concept clarity, career decision self-efficacy, and time pressure) and between the mediators (T2) and career decision-making stress (T2). We allowed the mediators to covary. Furthermore, we also included a direct path between condition (waitlist or intervention) and career decision-making stress (T2). All dependent variables in the model (the mediators and career decision-making stress) were regressed on their prior levels at the T1 measurement. This way we could more accurately estimate the change that coaching caused in the dependent variables. In addition, we estimated the significance of indirect paths from condition to career decision-making stress (T2) through the mediators using bias-corrected bootstrapping confidence intervals (10,000 samples; Hayes, 2009).

We tested whether the assumptions of linearity, homoscedasticity, normal distribution of residuals of the multilevel path model were met (Hox et al., 2017; Maas & Hox, 2004). We tested these assumptions at the individual level because all study variables were defined at this level.

## Results

### Participants

**Fig. 1:**
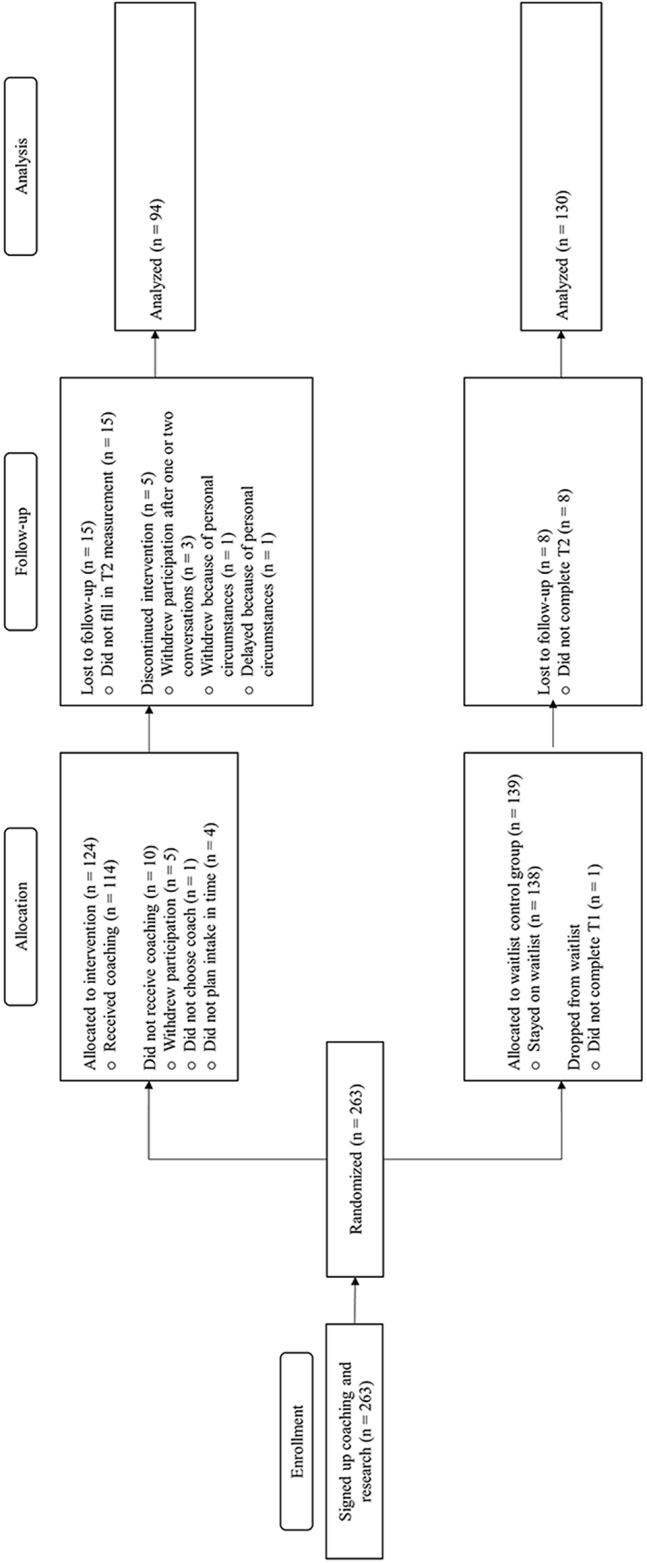
CONSORT flow diagram

A total of 263 participants enrolled in the study. The intervention group included 124 participants and the waitlist-control group 139. Participants dropped out of the study for several reasons (*n* = 39, see Fig. 1 for CONSORT flow diagram; Moher et al., 2001). The final sample consisted of 224 participants, 94 in the intervention group and 130 in the waitlist-control group. The average age of the participants was 22.85 (SD = 1.82) and 82.1% (*n* = 184) was female. In addition, we conducted a logistic regression analysis to assess whether the intervention and waitlist-control condition differed in terms of age and gender and our model variables measured at T1. We included age, gender, T1 self-concept clarity, T1 career decision self-efficacy, T1 perceived time pressure, and T1 career decision-making stress as predictors of condition. As the model was not statistically significant, χ2 (6, *N* = 223) = 9.69, *p* =.14, there were no significant overall differences between the intervention condition and the waitlist-control condition on these variables at the T1 measurement.

The average length of a coaching program was 316.74 days (*SD* = 64.21). The average number of clients coached by each coach was 13.43 (*SD* = 13.38). The median time between the T1 and T2 measurements was 341 days for the intervention group and 259 days for the waitlist-control group.

### Multilevel path modelling

Prior to testing the multilevel path model, we inspected whether inclusion of control variables was necessary. Only gender correlated significantly with condition (*r* =.15, *p* =.02), thus we included only gender as control variable. Furthermore, results of the assumption checks indicated that no assumptions were violated.

**Fig. 2:**
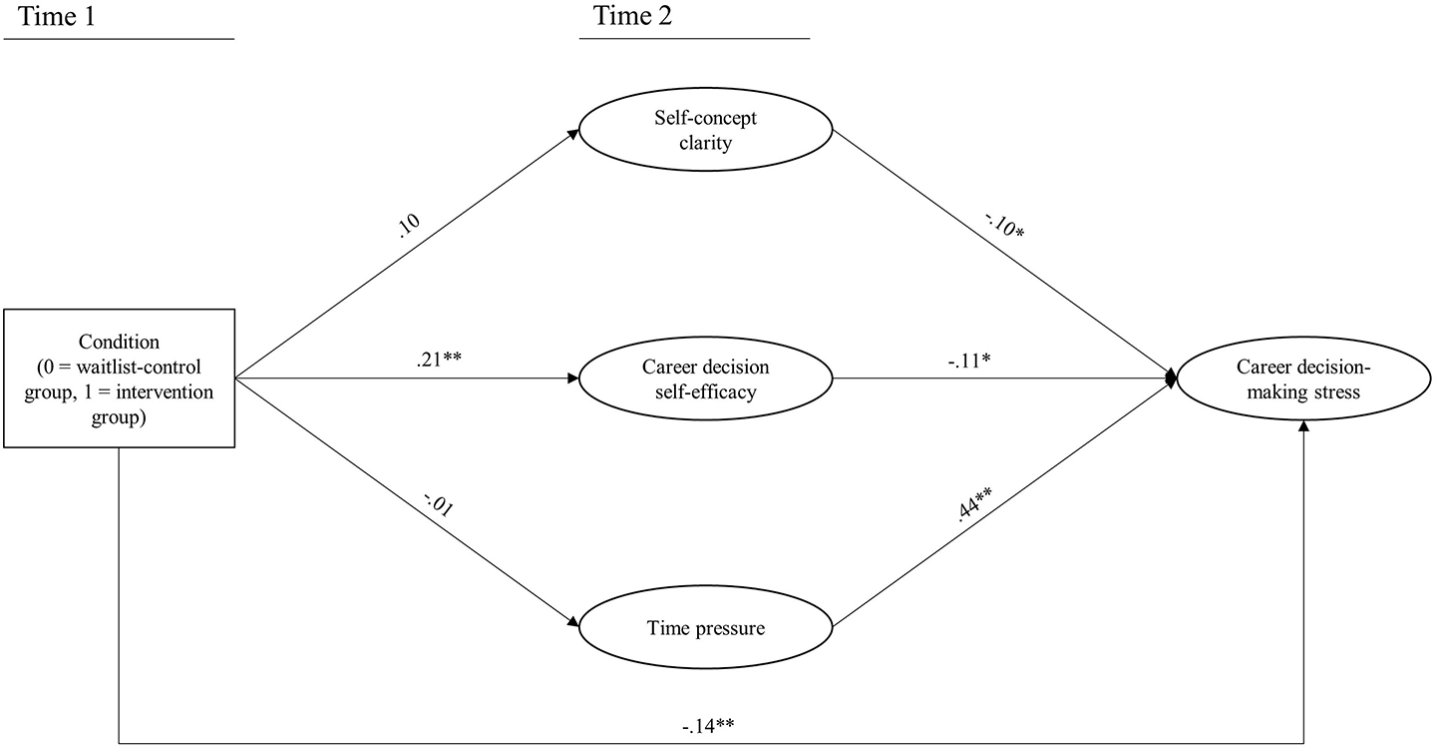
Graphical presentation results of the multilevel path analysis *Note* **p <.01 *p <.05. Standardized regression coefficients are reported. The full results of the multilevel path analyses can be found in Table 3

In testing the multilevel path model, consistent with Hu and Bentler’s (1998) prescriptions, we used the Standardized Root Mean Squared Residual (SRMR) and the Root Mean Square Error of Approximation (RMSEA) values, together with the Comparative Fit Index (CFI) values, to assess model fit. Favorable model fit is indicated by SRMR values of 0.08 or below, RMSEA values of 0.06 or below, and CFI values of 0.95 or above (Hu & Bentler, 1998). The fit of the tested multilevel path model was reasonable, χ2/df = 2.36, *p* =.002, SRMR = 0.07, CFI = 0.94, RMSEA = 0.08. Full results of the path analysis can be found in Table 3 and a graphical presentation can be found in Fig. 2.

**Table 3:** Results of the multilevel path model predicting career decision-making stress (*N* = 223)

Variables	Self-concept clarity (T2)			Career decision self-efficacy (T2)			Time pressure (T2)			Career decision-making stress (T2)		
	B	SE	β	B	SE	β	B	SE	β	B	SE	β
Condition^a^	0.14	0.08	0.10	0.28**	0.08	0.21**	-0.01	0.08	− 0.01	-0.25**	0.08	− 0.14**
Self-concept clarity (T1)	0.58**	0.05	0.65**									
Career decision self-efficacy (T1)				0.46**	0.10	0.47**						
Time pressure (T1)							0.26**	0.06	0.28**			
Career decision-making stress (T1)										0.29**	0.11	0.28*
Self-concept clarity (T2)										-0.12*	0.05	− 0.10*
Career decision self-efficacy (T2)										-0.15*	0.07	− 0.11*
Time pressure (T2)										0.50**	0.09	0.43**
Gender^b^										0.25	0.15	0.11
Intercept	2.21			2.95			3.18			1.65		
Residual variances	0.58			0.74			0.92			0.62		
*R* ^2^	0.42			0.27			0.08			0.38		

Note *p <.05, **p <.01. Unstandardized estimates (*B*), standard errors (*SE*), standardized coefficients (β) are presented. Abbreviations: ^a^0 = waitlist-control group, 1 = intervention group. ^b^0 = male, 1 = female. Model fit indices: χ2/df = 2.36, *p* =.002, SRMR = 0.07, CFI = 0.94, RMSEA = 0.08

Here we report relations between our focal study variables. Results demonstrate a significant negative total effect of coaching on career decision-making (-0.17; 95% CI [-0.31, -0.06]), reflecting a small to moderate effect size. Regarding the mediators, coaching had a significant and positive effect of condition on career decision self-efficacy (β = 0.21, *p* =.001). Students in the coaching group felt more self-efficacious regarding their career decision after their coaching program compared to participants in the waitlist-control group. However, the effects of condition on self-concept clarity and time pressure were non-significant (β = 0.10, *p* =.08 and β = − 0.01, *p* =.95, respectively). The relationships between the mediators and career decision-making stress were significantly negative for self-concept clarity (β = − 0.10, *p* =.03) and self-efficacy (β = − 0.11, *p* =.04), and significantly positive for time pressure (β = 0.43, *p* <.001). Finally, the direct relation between condition and career decision-making stress (i.e., over and above the mediators) was significant (β = − 0.14, *p* =.004).

In a next step, we assessed the standardized bias-corrected bootstrapping confidence intervals of the indirect effects of condition on career decision-making stress through the mediators. The indirect effects through self-concept clarity and time pressure were non-significant (-0.01; 95% CI [-0.04, 0.00] and − 0.00; 95% CI [-0.06, 0.07], respectively), while the indirect effect through career decision self-efficacy was significantly negative (-0.02; 95% CI [-0.06, -0.00]). In sum, the coaching intervention reduced career decision-making stress both directly, and indirectly through its positive effect on career decision self-efficacy.

### Exploratory analyses

We explored whether the effectiveness of the coaching intervention depends on students’ initial attitude toward coaching at T1. In addition, we explored whether students changed their career choices between the pre- and post-measurement (T1 and T2), and whether this differed between students in the coaching and control condition.

#### The moderating role of coaching attitude

We tested whether coaching attitude (T1) moderated the relation of condition (i.e., coaching vs. control) with self-concept clarity, career decision self-efficacy, and time pressure (the proposed mediators measured at T2). Before the analyses, we mean-centered coaching attitude and created an interaction term (condition x coaching attitude). For reasons of power and multicollinearity, we tested the moderating role of coaching attitude for each mediator separately. Specifically, we tested three separate models in Mplus based on our original research model, with added paths from coaching attitude and the interaction term to the respective mediator.

The first model shows that coaching attitude did not relate to self-concept clarity (β = 0.05, *p* =.51), but moderated the relation between condition and self-concept clarity (β = − 0.13, *p* =.02). The simple slope of condition on self-concept clarity was significantly positive at low levels (*M*– 1 *SD*) of attitude toward coaching (*B* = 0.26, 95% CI [0.12, 0.41]), and non-significant at high levels (*M* + 1 *SD*) of coaching attitude (*B* = -0.00, 95% CI [-0.22, 0.21]. This indicates that coaching was effective in clarifying the self-concept of students who initially had a relatively negative attitude toward coaching but not for students with a relatively positive coaching attitude. In addition, the second and third models showed that, coaching attitude was not related to career decision self-efficacy (β = 0.12, *p* =.21) and time pressure (β = − 0.00, *p* =.96) nor did it moderate the relations between condition and career decision self-efficacy (β = − 0.11, *p* =.15) and condition and time pressure (β = 0.01, *p* =.89).

#### Changes in career choices and career choice certainty

We qualitatively assessed whether students changed their career choices between the pre- and post-measurement (T1 and T2), and whether this differed between students in the coaching and control condition. First, we created a data file containing the verbatim descriptions of participants’ career choices on the pre- and post-measurement (examples of career choices are: general practitioner, pediatrician, surgeon etc.). We developed a protocol for assigning a score to the difference between students’ career choice at T1 and T2 (see the scores 0 to 5 in Table 4). Then, three authors independently rated the difference between career choice T1 and T2 for each student and reached an agreement of 82.1%. Based on the disagreements between the raters, two other authors added an additional scoring category to the protocol (score 6). Finally, the three authors again independently scored the difference between the T1 and T2 career choices. This resulted in an agreement rate of 93.8%. The fourteen remaining disagreements were discussed amongst the three authors and agreed upon. Table 4 presents the frequencies of each category per condition.

**Table 4:** Results of qualitative analyses of career choice changes

			0. No career choice change	1. Change from one to anothter career choice	2. Career choice specification^a^	3. From indecision to a career choice	4. From indecision to indecision	5. Further specification within specialism^b^	6. Broadening of career choice^c^	Total
Condition	Waitlist control	Frequency	65	40	9	9	2	1	4	130
		% within condition	50.0%	30.8%	6.9%	6.9%	1.5%	0.8%	3.1%	100.0%
	Intervention	Frequency	30	45	8	7	1	1	2	94
		% within condition	31.9%	47.9%	8.5%	7.4%	1.1%	1.1%	2.1%	100.0%
Total		Frequency	95	85	17	16	3	2	6	224
		% within condition	42.4%	37.9%	7.6%	7.1%	1.3%	0.9%	2.7%	100.0%

Note ^a^From multiple career choices at T1 specified to one career choice at T2. ^b^E.g., from surgeon at T1 to cardiothoracic surgeon at T2. ^c^More options mentioned at T2 compared to T1

The categories reflect: no change in career choices between T1 and T2 (category 0; e.g., from “pediatrician” to “pediatrician), a change from one career choice to another (category 1; e.g., from “oncologist” to “general practitioner”), a career choice specification (category 2; e.g., from “general practitioner, internist, or psychiatrist” to “general practitioner”), a change from indecision to a career choice (category 3; e.g., from “I don’t know” to “internist”), indecision on both T1 and T2 (category 4; e.g., from “I don’t know” to “I don’t know”), a further specification within a specialization (category 5; from “internist” to “oncologist”), and the broadening of a career choice (category 6; e.g., from “plastic surgeon” to “plastic surgeon, anesthesiologist, intensive care specialist, or cardiologist”).

Next, we quantitatively assessed whether condition (control vs. coaching) was associated with career choice changes between T1 and T2, that is, whether students in the coaching condition were more likely to change their career choices (between T1 and T2) than students in the control condition. Further, we explored whether condition, career choice changes, and the interaction between the two related to students’ career choice certainty at T2. For the purpose of these exploratory analyses, we created a new variable reflecting career choice change (0 = no, 1 = yes). The ‘no’ category (Table 4, category 0) was left as is. For the ‘yes’ category we merged the scores one, two, three, and five (see Table 4) to reflect career choice change into the direction of a more specific career choice at T2. We left out the scores four and six (4% of the sample) as no career choice was specified at T2.

To assess whether coaching was associated with career choice changes between T1 and T2 we performed a logistic regression analysis. We included condition as predictor of career choice change and added gender as control variable (consistent with the main analyses). The model was statistically significant, *χ*^*2*^ (2, *N* = 224) = 9.38, *p* =.009. Condition was significantly related to career choice change, Exp(*B*) = 2.33, *p* =.004, indicating that (controlling for gender) students in the coaching condition were 2.33 times more likely to change their career choice than students in the control condition.

Additionally, we performed a hierarchical regression analysis to assess whether and how condition, career choice change, and their interaction were associated with career choice certainty at T2. In the first step, we included the control variables (gender and career choice certainty at T1), condition, and career choice change as predictors of career choice certainty (T2). In the second step, we added the interaction term (condition x career choice change) to the model. The model in the first step was statistically significant, *R*^2^ = 0.25, *F*(4, 201) = 16.36, *p* <.001. Condition was significantly related to career choice certainty (β = 0.21, *p* =.001), indicating that coaching led to more career choice certainty. That is, controlling for gender, students in the coaching condition became more certain about their career choice than students in the control condition. The relation between career choice change and career choice certainty was not significant (β = − 0.12, *p* =.07). The addition of the interaction term (condition x career choice change) in step two did not explain additional variance in career choice certainty (Δ*R*^2^ = 0.001, Δ*F* = 0.26, *p* =.61) and the interaction term was not related to career choice certainty (β = 0.06, *p* =.61).

We conclude that, compared to students in the waitlist-control condition, more students in the coaching condition changed their career choice and that students in the coaching condition became more certain about their (possibly changed) career choice.

## Discussion

This study investigated whether and through which mechanisms coaching is effective in reducing career decision-making stress among medical students. Using a randomized controlled-trial design, we found that coaching is effective in reducing career decision-making stress through increasing career decision-making self-efficacy. Coaching increased students’ confidence in their ability to complete career choice related tasks which was associated with less career decision-making stress.

As predicted, we found that coaching increases medical students’ career decision-making self-efficacy and in line with prior research (Fris et al., 2022), career decision self-efficacy was negatively associated with career decision-making stress. The students in the intervention group were more confident in their ability to complete career choice related tasks than those in the waitlist-control condition. While we reasoned that verbal persuasion and reflection on mastery experiences (Bandura, 1977; Behrendt et al., 2021; Van Zyl, 2016) will enhance career decision-making self-efficacy, we were unable to test coaches’ supportive behaviors in this regard. We encourage future research to utilize a longitudinal experience sampling design and measure coach and client perspectives to deepen our understanding of the specific coaching processes that support the development of career decision-making self-efficacy.

Prior coaching studies have found that coaching can stimulate self-reflection and increase self-insight (Grant, 2003; Rank & Gray, 2017). However, our results show no overall main effect of coaching on students’ self-concept clarity. We expected that coaching would stimulate self-reflection among students, clarifying and stabilizing their beliefs about themselves. A potential explanation for this null finding is that the self-concept clarity construct is too broad of a measure in this context. It is possible that coaches focused more directly on fostering attainment of students’ career goals. As a result, coaching may have affected a more specific self-concept, for example students’ vocational identity (i.e., clarity of students’ career goals, interests, and talents; Holland, 1997). Similarly, Dik and Steger (2008) found stronger effects of a career workshop on a career-specific outcome than on a more global outcome.

However, exploratory analyses demonstrated that coaching clarified the self-concept of students with a relatively negative coaching attitude before coaching. Possibly, students’ less positive attitude toward coaching reflects lower openness to new experiences in general, which is associated with lower engagement in self-reflection (Trapnell & Campbell, 1999). Coaches may have particularly stimulated self-reflection in students who are generally less inclined to reflect on themselves. In line with prior research on the relation between the clarity of vocational self-belief and career decision-making stress (Praskova et al., 2015), self-concept-clarity was negatively associated with career decision-making stress. Therefore, future research could (experimentally) investigate which techniques coaches can best use to help clients reflect on their self-concept.

Our results demonstrated that perceived time pressure was associated with more career decision-making stress, but that coaching did not reduce students’ perceived time pressure. Potentially, coaches in this intervention may have paid less attention to strengthening students’ time management and planning skills, which may explain our null-finding. Additionally, time pressure is inherent in the medical school curriculum and the transition from school to work, and as such difficult for students to control. Therefore, the perceived time pressure associated with career decision-making stress may be inherently challenging to influence, even through personalized interventions like coaching. Future research could examine whether perceptions of time pressure can be alleviated if coaches pay more specific attention to this impactful factor, for example, by providing students with tools to manage their cognitions about time.

Our exploratory analyses revealed that the students in the coaching group changed career choices more often and became more certain of their career choices than students in the waitlist-control group. These outcomes show that coaching can have a substantial impact not only on students’ career decision-making stress but also on their actual career decisions. Coaching may have provided a safe setting in which students could critically evaluate their current career choice and its alignment with their interests, strengths, values, and career preferences. Future research could examine whether the quality of students’ career choices (e.g., person-job fit after graduation) also improves because of coaching.

### Limitations and suggestions for future research

While the use of a randomized waitlist-controlled trial design allowed for drawing causal conclusions on the effects of the coaching intervention, some limitations should be taken into account when interpreting our findings. The tailored individual approach is key to coaching (Grant, 2003; Theeboom et al., 2017), but simultaneously poses challenges when it comes to the replicability of research findings. Future research could measure which coaching techniques and approaches are used by coaches and evaluate their effectiveness. In addition, studies could focus on whether the effectiveness of coaching approaches and techniques is dependent on the type of coaching goal or the characteristics and preferences of the client (e.g., need for cognition; Cacioppo et al., 1996). In addition, our sample consisted of students who voluntarily signed up for coaching, which limits the generalizability of our findings to the broader medical student population. However, since coaching inherently focuses on supporting individuals in achieving self-selected goals, which requires a certain degree of voluntariness, our sample may be representative of the population likely to engage in coaching.

Another potential limitation of this study is the general context in which it took place. Data were largely gathered during the COVID-19 pandemic, which may have impacted the levels of career decision-making stress of students (Fris et al., 2024). For example, the coaching intervention might only be effective when career decision-making stress levels are relatively high. Future research could investigate whether contextual factors affect the effectiveness of coaching in reducing career decision-making stress. Similarly, the study could be replicated in different hospitals and additional vocational contexts to increase generalizability of our findings. Hospitals and other master’s programs may for example differ in levels of competition and supervisor support, which may affect coaching effectiveness.

The time between the first and second measurement was longer for the intervention group compared to the control group, potentially biasing the findings through maturation. As students progress through their studies, they may become more self-efficacious regarding their career choices and as a result experience less career decision-making stress. However, our findings suggest that a maturation bias is less likely because career decision self-efficacy of participants in the waitlist-control condition did not change over time whereas career decision self-efficacy in the intervention condition increased (see means in Table 1). Also, career decision-making stress slightly increased in the waitlist-control condition, whereas it slightly decreased in the intervention condition. Still, future RCT studies in natural settings could address a possible measurement gap between the intervention condition and control groups. Researchers could randomly split up the control group in smaller groups and match the timing of Time 2 measurements based on the number of participants that finished their coaching. This way, a difference in the timing of measurement can be decreased and conclusions can be drawn with even more confidence. For ethical reasons, we could not extend the waiting time of the control condition. Therefore, we cannot draw conclusions on the longer term effects of coaching.

We measured the mediating variables and the outcome variable at the same point in time with the same method (i.e., self-report questionnaires). Hence, common method variance may have increased relations between the mediating and outcome variables measured (Podsakoff et al., 2003; Podsakoff & Organ, 1986). In addition, self-reports are prone to social desirability bias (Podsakoff et al., 2003). We minimized these biases by varying our scale anchors and by ensuring anonymity of responses (Podsakoff et al., 2003). Although outcomes such as self-concept clarity, self-efficacy, and career decision-making stress are best measured by asking the respondents themselves, future research could additionally include non-self-report measures such as coach perceptions of clients’ progress. However, it is important to consider that coaches’ perceptions of their clients’ progress may also be subject to bias.

Additionally, our measurement-of-mediation design (i.e., measuring the mediators rather than manipulating them) limits our ability to draw robust conclusions about the psychological process through which coaching influences career decision-making stress (Spencer et al., 2005). As we did not manipulate the mediating variables, we cannot rule out reverse causality. For example, a reduction in career decision-making stress could result in higher career decision self-efficacy instead of the other way around. However, when manipulation of the mediators is difficult, as in this study, our choice of design is recommended (Spencer et al., 2005). Future research could temporally separate the measurement of the mediators and outcome variables to provide more evidence for the working mechanisms of coaching.

A final limitation of this study is the possible selective attrition in the intervention condition. Several students did not complete the Time 2 measurement (*n* = 15). However, a logistic regression analysis showed that the attrition was likely to be random as Time 1 levels of career decision-making stress, self-concept clarity, career decision self-efficacy, and time pressure were not related to completion of the T2 measurement, *χ*^*2*^ (4, *N* = 109) = 0.19, *p* >.99. The dropout rate in the waitlist-control condition may have been less because these participants could start their coaching program after completing the second questionnaire.

An interesting finding was that the direct effect of coaching on career decision-making stress remained significant when controlling for the mediating mechanisms. This suggests that additional mechanisms, not measured in this study, are at play. For example, coaching could diminish career decision-making stress through stimulating goal-directed behaviors. While students’ values, motives, and interests can be explored during coaching sessions, part of their reflection may also occur in between coaching sessions (e.g., during clinical clerkships). In fact, it is important that students are encouraged to remain active exploring their career thoughts and career options (Kleine et al., 2021) after the coaching sessions. The extent to which a coach succeeds in initiating this cognitive reflection and environmental exploration could have a significant influence on the extent to which students are able to make a well-considered career choice and thus reduce their career decision-making stress. The medical background of the coaches in this study may have been valuable in helping students explore their environment, as the coaches were already familiar with it. Future research could empirically test these and other mechanisms explaining the effectiveness of coaching in reducing career decision-making stress.

Future research could also investigate whether coaching interventions for medical students are cost-effective. For example, coaching of master students may reduce dropout in subsequent career stages (i.e., among residents), which would be very valuable. During coaching, students are encouraged to make informed career choices that fit their abilities and needs, which will reduce this costly dropout. Our exploratory findings indicate that coached students reconsidered their career choice and became more certain of their choices. This indicates that they actively explored career alternatives and chose a better fitting career option. Future research could investigate the quality of these choices and how it affects residency dropout. Also, the cost-effectiveness of coaching can be examined in relation to alternative career interventions (e.g., career workshops; Dik & Steger, 2008). Another important research question is whether the effects of coaching may differ depending on participant characteristics. In the present study, we found some indications for such moderating effects for attitude towards coaching. However, future research is needed to examine other moderating factors. Such insights can assist medical programs to target individuals for whom coaching is most beneficial, which benefits the cost effectiveness of coaching.

### Implications for medical education

This study demonstrates that coaching can be an important tool to support medical students’ career decision-making and to reduce their levels of career decision-making stress. Of all career interventions, coaching is the most personal and tailored to individuals’ needs. Such individualized support is crucial to the effectiveness of career choice interventions (Whiston et al., 2017). At the same time, the costs of coaching are high and it is unlikely that medical schools can provide coaching to all students. Therefore, medical schools could develop career development programs that include more scalable, evidence-based interventions for all students (e.g., two-session workshops and group-coaching; De Lasson et al., 2016; Van der Horst et al., 2021) combined with more individualized interventions like coaching. More scalable interventions can be integrated in the medical curriculum and thus provided to all students. Coaching can be optionally provided to students in need of extra career support. This way, all students get the career support they need, without unnecessary use of resources. Moreover, integrating a smaller-scale coaching program into broader career development initiatives enhances the feasibility of recruiting (physician) career coaches.

### Conclusion

In conclusion, career decision-making stress is prevalent among medical students (De Geneeskundestudent, 2019) and is associated with negative outcomes such as career indecision and lower psychological well-being (Arslan & Bayraktar-Uyar, 2020; Creed et al., 2016; Germeijs et al., 2006). Our study demonstrates that career coaching is an effective intervention to reduce this stress and to support students in their career decision-making through increasing career decision self-efficacy. Therefore, medical programs could consider providing coaching to medical students during their master’s program and clinical clerkships. Besides fostering students’ well-being, providing such career support can help them to make career decisions that best suit their preferences, values, strengths, and motivation resulting in higher job satisfaction, well-being, and performance later in their careers (Kristof-Brown et al., 2005).

## Electronic supplementary material

Below is the link to the electronic supplementary material.

Supplementary Material 1

## Data Availability

To ensure the protection of privacy and confidentiality of the participants, supporting data is not available.
