# Supplementary material for: Career coaching to support medical student career decision-making: a randomized controlled trial
Source: Adv Health Sci Educ Theory Pract. 2025 Mar 3;30(5):1497–521. doi: 10.1007/s10459-025-10409-8 (PMC12572062; doi:10.1007/s10459-025-10409-8)
Supplement: Supplementary file 1 — Supplementary Material 1 [file 10459_2025_10409_MOESM1_ESM.docx]

Online Resource 1 – Complete List of Items

Self-concept clarity^a^ (1 = strongly disagree, 5 = strongly agree)

- My beliefs about myself often conflict with one another. (R)
- My beliefs about myself seem to change very frequently. (R)
- In general, I have a clear sense of who I am and what I am.
- Even if I wanted to, I don’t think I could tell someone what I’m really like. (R)

Career decision self-efficacy (1 = strongly disagree, 5 = strongly agree)

I am capable of…

- …determining what my ideal job would be.
- ….making a career decision I support.^b^
- …choosing a career that will fit my preferred lifestyle.

Time pressure (1 = not applicable at all, 5 = fully applicable)

- I experience time pressure in my search for a career direction that suits me.
- I have enough time to find out which career direction suits me. (R)
- I need to determine a direction in my career quickly.

Career decision-making stress (1 = never, 5 = often)

In the past 4 weeks…

- … I was worried about choices I have to make in my study or career.
- … I worried about the direction I want to take in my career.
- … I felt nervous about important decisions I need to make regarding my future work.
- … I felt stressed about having to make important career choices.

Coaching attitude (1 = strongly disagree, 5 = strongly agree)

- I am skeptical about the usefulness of coaching. (R)
- Medical students who are coached will benefit greatly.
- A coach can help medical students to develop themselves.

Career choice and career choice certainty

- If you had to choose a specialization and/or further education/career right now, what would you choose? (open-ended question)
- How sure are you about that choice? (answers coded as 1 = very unsure, 5 = very sure)

(R) = reverse coded item

^a^Originally, the self-concept clarity scale contained five items. We noticed that the content of the item “I spend a lot of time wondering about what kind of person I really am” (R) did not necessarily reflect self-concept clarity but might be more related to engaging in self-reflection. Removal of this item increased Cronbach’s alpha from .69 to .76. Therefore, we excluded this item in the further analyses.

^b^This item was adjusted to the Dutch context in line with the definition of career decision self-efficacy.
